# Shared Purpose: Leveraging a Community-Academic Partnership to Increase Local Environmental Health Awareness via Community Science

**DOI:** 10.35844/001c.38475

**Published:** 2022-10-24

**Authors:** Lisa J. Martin, Vincent Hill, Curtis Maples, Theresa Baker, Shereen Elshaer, Melinda Butsch Kovacic

**Affiliations:** 1 Department of Pediatrics, Cincinnati Children’s Hospital Medical Center; University of Cincinnati College of Medicine,; 2 University of Cincinnati,; 3 Seven HIlls Neighboorhood Houses,; 4 Pediatrics, Cincinnati Children’s Hospital Medical Center,; 5 Department of Pediatrics, Cincinnati Children’s Hospital Medical Center; Department of Public Health and Preventive Medicine, Mansoura University Faculty of Medicine,; 6 Cincinnati Children’s Hospital Medical Center, Department of Pediatrics, University of Cincinnati College of Medicine; Department of Rehabilitation, Exercise, and Nutrition Sciences, the University of Cincinnati College of Allied Health Sciences

**Keywords:** community-academic partnership, community science, health promotion education, urban environmental health

## Abstract

Environmental factors can lead to disease and health disparities when the places where people live, learn, work, play and pray are burdened by social inequities. Non-formal programs that explicitly connect local environmental exposures and human health could be of great value to communities at greatest risk. The purpose of this work was to co-create relevant and engaging education with youth and community stakeholders of all ages that more explicitly emphasizes the link between the local environment and community members’ health through a hands-on community science experience. Our experiences helped strengthen our community-academic partnership and establish a route to create and tailor informal programming to meet local needs *and* engage people in community science with academic partners. We generated two distinctly different community science neighborhood audit tools designed to differently engage our community partners and inform community participants of their local environments and its role on their health. Through community meetings, we garnered critical insight from our stakeholders. While neither of the tools and accompanying data collected were deemed to be scientifically generalizable, our ongoing and future work has benefited from important lessons learned from their creation and sharing.

## Introduction

Environmental factors such as air and water pollution are fundamental determinants of health and well-being (Bein et al., 2022; Hautekiet et al., 2022; Heydari et al., 2022; National Research Council (U.S.) & Institute of Medicine (U.S.), 2013). Importantly, environmental factors can lead to disease and health disparities when the places where people live, learn, work, play, and pray are burdened by social inequities (Beck et al., 2013; Braveman & Gottlieb, 2014; Chesney & Duderstadt, 2022; HealthyPeoplegov, 2019). If present in schools’ curriculum at all, environmental education often aims to increase public awareness and knowledge about global environmental issues such as climate change. While the NIH (National Institute of Environmental Health Sciences, 2020) and the EPA (United States Environmental Protection Agency, 2021) have online curricula, rarely is it paired with human health education in schools (aka environmental health) (Keselman et al., 2011). Further, good environmental knowledge alone does not necessarily translate into pro-environmental behaviors in youth (Liu et al., 2020; Maipas et al., 2021; Naustdalslid, 2011; Trott, 2021). With the barrier of time in the school curriculum, more non-formal programs that explicitly connect local environmental exposures and human health, such as those experienced by persons with asthma, could be of great value (Davis et al., 2018; Maipas et al., 2021). By including human health in such programs and debating the impact of environmental factors on people “like me,” an individual can relate their contributions to local and global environmental challenges.

The implementation of combined human and environmental health programs requires community-specific considerations, particularly as communities have different health risks and environmental difficulties. Thus, addressing these challenges in a relevant social, economic, and political context may be the most impactful (Ramírez et al., 2015; Sandhaus et al., 2018). Engagement of the public in scientific research (aka community or citizen science) provides scientists with community context while also communicating the health risks associated with environmental exposures to non-professional scientists living in the community (Bonney et al., 2014). Success can be maximized by engaging experts from various scientific disciplines, community partners, citizens from affected communities, as well as other stakeholders (Stokes et al., 2010). A powerful approach to bringing together diverse individuals is the community-academic partnership (Drahota et al., 2016; Fields et al., 2021). Unlike traditional academic research where the solutions are determined by scientific professionals, in community-partnered work, the community participates in determining the focus and aids in project development, providing the opportunity to capture authentic relevant data (Israel et al., 2005). Thus, community-academic partnerships are critical elements to co-creating relevant and engaging programs that lead to systematic high-quality data collection, greater awareness of environmental health challenges, and solutions.

Citizen science engages community participants to collect and record local observations, contextualize data with local knowledge, and discuss how the local environment impacts their own community’s health (Bonney et al., 2015). The purpose of this work was to co-create relevant and engaging education with youth and community stakeholders of all ages that more explicitly emphasizes the link between the local environment and community members’ health through a hands-on community science experience. It was expected that the resulting data could be used by scientists and partnering communities alike to better understand the critical connection between the two. Indeed, the actualization of knowledge to behavior is associated with increased self-efficacy (Kulik et al., 2019; Phoosuwan & Lundberg, 2020) and provides opportunities for pro-community-wide behavior changes and advocacy (English & Baldwin, 2020; Khatibi et al., 2021). To this end, in Summer 2016, we began weekly meetings with members of the West End community in Cincinnati, Ohio, to discuss issues related to their community’s environmental health. Together, we co-designed and piloted an environmental audit to capture data to fuel our discussions. Following the audit, we considered our lessons learned and communicated our results with members of the neighborhood’s community council. A written report was shared broadly. In Summer 2019, a member of this group reinitiated conversations about our shared effort as a paid undergraduate summer research fellow. The fellow chose to create and test a reiteration of the original environmental audit tool for broader use by non-professional community scientists in his neighborhood and beyond. The new tool incorporated many of the lessons learned from the earlier discussions and led to many new lessons. This article is a summary of these collective efforts. It exemplifies the value of community-academic partnerships in the co-creation of non-formal community-focused environmental health programming using community science and details the directions we are heading for our future program development.

## Methods and Results

### The Partnership

We began our community-academic partnership in 2011, intending to build trust and create opportunities for mutually beneficial education and research efforts (Kovacic et al., 2014). While the approaches used in community-academic partnership can be varied (Drahota et al., 2016), our partnership was established using community-based participatory research (CBPR) collaborative approach. In this approach, all partners—particularly those most affected by the research—are involved in the research process. As a result, researchers are challenged to listen to, learn from, solicit, and respect the contributions of, and share power, information, and credit for accomplishments with groups or communities that they are targeting (Wallerstein & Duran, 2010).

**Figure 1.**
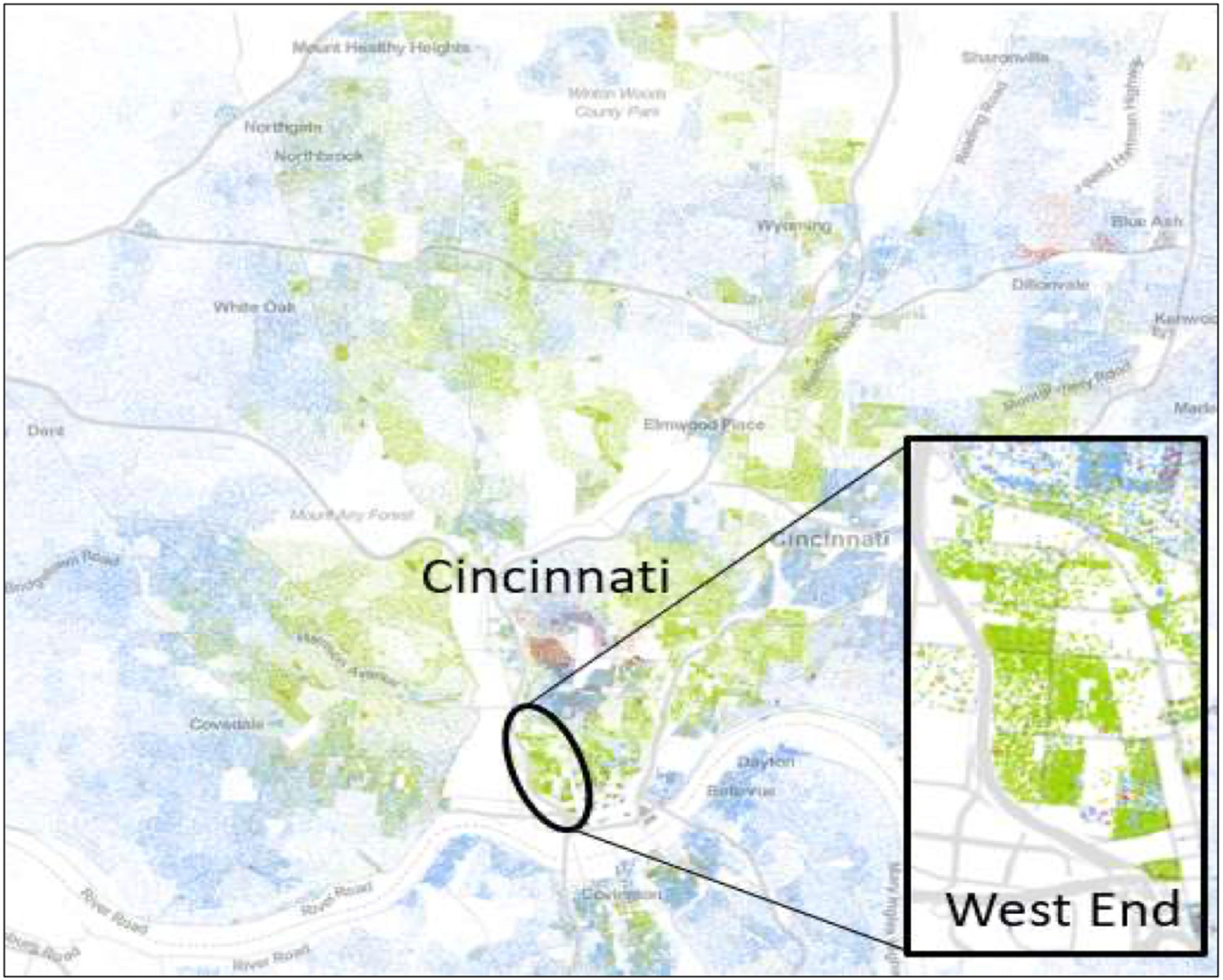
Distribution of Blacks (green dots) and Whites in Cincinnati (blue dots) (Cable, 2013).

Our community-academic partnership was between Seven Hills Neighborhood Houses in the West End and researchers from Cincinnati Children’s Hospital/the University of Cincinnati. We partnered with the Seven Hills Neighborhood Houses due to our established relationship, having had multiple institutionally-sponsored grants and more recently a federally-sponsored grant as well as the importance of the Seven Hills Neighborhood Houses to the West End neighborhood that it serves. Indeed, the West End is an urban neighborhood in Cincinnati, Ohio, with a population of more than 6,500 residents as of the 2010 Census (City of Cincinnati, 2019). The population residing in Cincinnati’s West End is predominantly Black/African American [84.3%] (see Figure 1). Many of the Black/African Americans in Cincinnati have ties to this region as it has been a primarily Black/African American neighborhood since the 1920s. Approximately 51% of the households live below the poverty line, and 33% lack a high school diploma (City of Cincinnati, 2019). Further, because the neighborhood is located along an interstate highway corridor, residents are exposed to high levels of traffic-related air pollution in addition to pollution from numerous manufacturing plants (Ryan & LeMasters, 2007).

The West End is also a neighborhood with a strong sense of community. Seven Hills Neighborhood Houses is a social services agency serving the West End since 1961. It seeks to improve the quality of life in the community and serves approximately 2,500–3,000 at-risk children, teens, families, seniors, and disadvantaged community members annually. Services include: Advocacy (state-funded Victims of Crime Advocacy and Trauma Recovery Center); Basic Needs and Support (including food and household pantry); recreation and arts; health and nutrition (Health Rhythms drumming, Findlay Street Café to ensure residents meet nutritional needs, a Community Research Advisory Board); education (Sister Link Women’s support group and Distant Learning Center); and, in more recent years, community development and housing.

### Motivation for Using Community Science

The Greater Cincinnati region is committed to Open Data, broadly sharing information on a broad number of categories (oki.org/mapsapps/). So, some may ask, why have people collected data? Why not use the environmental apps that are available? The answer is about encouraging more people to become advocates and to see their communities for themselves—sometimes for the first time. People often fail to notice environmental factors that positively and negatively impact their health. Many community members likely accept their environment as it is. Advocates can see and advocate for healthier environments both for the sake of their environments and their community members’ health. Indeed, we (MBK) previously partnered with Seven Hills Neighborhood Houses on a PhotoVoice study where we asked participants to represent their perspectives through photographs to capture information about issues of importance to them (Hergenrather et al., 2009). The study concluded that participants often did not notice the waste and pollution surrounding them (Kovacic et al., 2014). Further, participants did not connect how poor environmental conditions may lead to chronic health issues. We reasoned that an educational environmental health program to make participants aware of the impact of local environmental factors on their community’s health would help residents become more aware of the problems and enable them to better advocate for change.

Further, the community council was supportive of such an endeavor as it was recognized that a poor environment also limited economic development. They had envisioned a “Clean Team” who would focus on cleanliness and safety in the West End. Indeed, as part of the West End’s quality of life planning, “clean” was identified as one of the seven pillars needed for community development (City of Cincinnati, 2016). As part of the educational programming, it became clear that data was needed to fuel conversations and substantiate the need for resources to improve the cleanliness of the community, and so began the co-creation of an audit tool to systematically allow participants to personally evaluate their local environments and provide data for the group’s discussions. With the support of professional scientists, the data also could be used to support scientific endeavors. By sharing the effort’s results with critical community members, the group could promote environmental health broadly as well.

### Early Audit Tool Development Creation and Use

#### Phase 1

As the first step in tool development, MBK partnered with a community volunteer (CM) in 2016. Together, their goal was to create a program to encourage environmental health exploration via community science in the West End. Specifically, the goal was to promote discussions of the effects of an unclean, polluted environment on physical, emotional, mental, and social health and on the neighborhood economy. Prior PhotoVoice studies supported the need for education. Still, the community volunteer highlighted the need to hear the community’s thoughts about the major factors going into the tool and create an open dialogue. It was decided that the program would be spread over multiple weekly meetings held at Seven Hills Neighborhood Houses. Upon review, the program was considered Not Human Subject’s (NHS) research by Cincinnati Children’s Institutional Review Board. Each meeting lasted roughly an hour and a half. The program was advertised at the community center through a flyer and community leaders encouraged participation. Individuals who had previously interacted with MBK and CM were invited to participate, including the “Clean Team” community leader. A total of 14 individuals (including the two facilitators who were a researcher (MBK) and a community volunteer (CM)) agreed to participate in the six-week program, composed of 11 males and three females (see Table 1). Most participants were African American (n=11), with three white participants. The age of the participants ranged from 11 to 72 years and education varied from less than a high school degree to a completed college degree. Participants were either West End residents (n = 10) or West End stakeholders (n = 4).

Prior to the initial meeting, MBK and CM outlined the proposed content. At the first meeting, CM led a discussion about the definition of environmental health. After the participants had gained a general understanding of environmental health, there was a discussion about what topics should be covered (see Table 2) and the desired number of meetings. It was agreed that there would be six meetings. The next meeting would include a discussion about how environmental conditions could impact residents’ health and the local economy, and how community science could be used to collect information to help the group better understand the association between the environment and health. Thereafter, the participants opted that the remaining meetings would be focused on activities leading to the creation and implementation of an environmental audit tool. While in this paper, we refer to the joint activities in developing the tool as co-design, the program leaders (MBK and CM) were not familiar with this approach at the initiation of the program. Rather, the desire to include the participants in the tool’s design came about organically, based on the desire to have data that would empower the community to understand better the environmental health concerns being overlooked every day while also collecting scientifically usable data.

An important part of the program was the educational component as our prior interactions with community members had shown a lack of recognition of environmental health. The community volunteer (CM) suggested an informal setting where the focus was more on discussion than the traditional lecture. Prior to each meeting, the program leaders prepared two to three questions for discussion.

**Figure 2.**
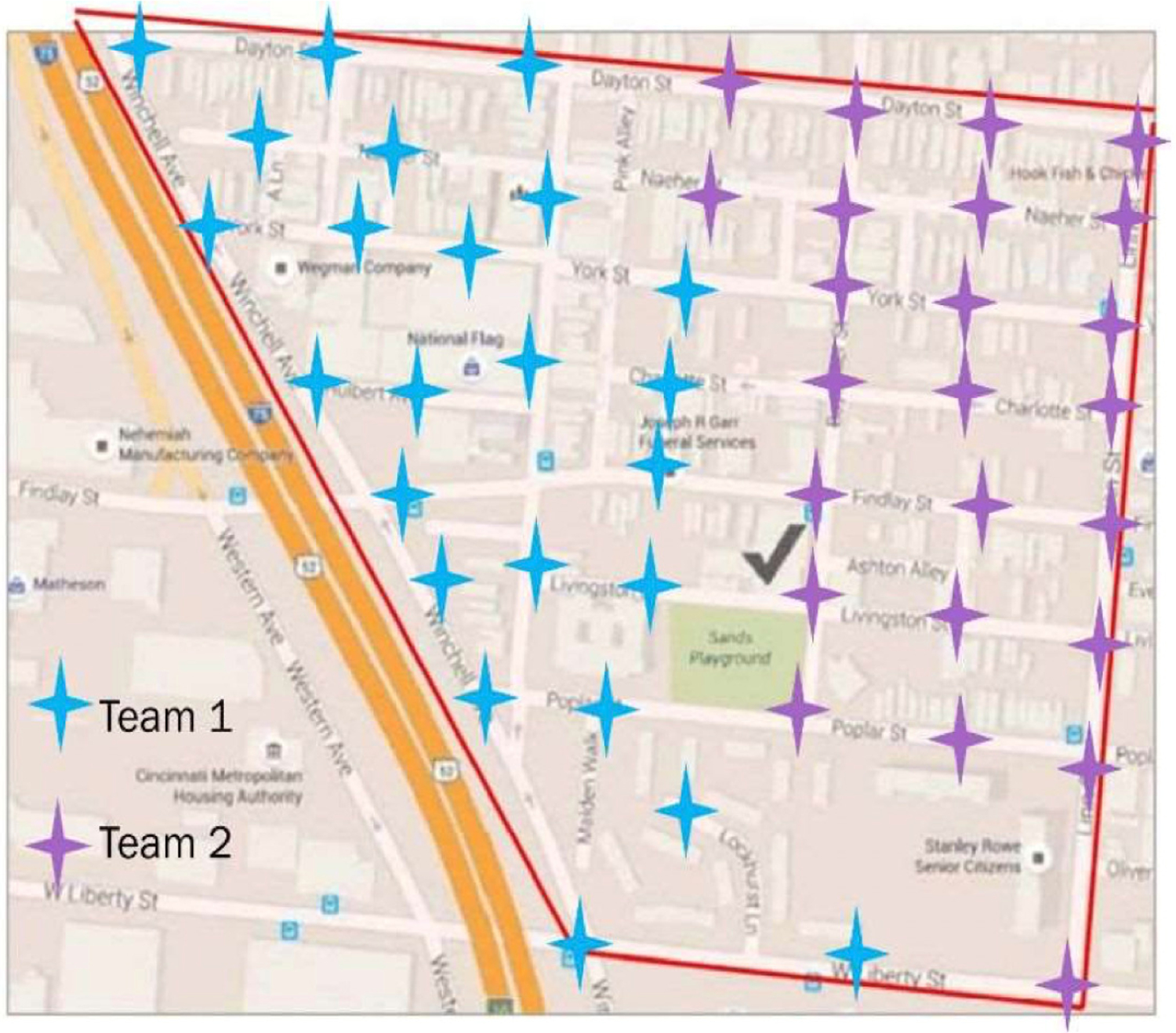
Phase 1 Observation Planning Map.

In preparation for creating the audit tool, the participants identified four areas of highest concern: cleanliness/pollution, traffic, neighborhood attractiveness/usefulness, and people/safety. Questions to be included in the future audit tool were developed for these topic areas and reviewed by the group. The final audit had eight to ten questions/observations per topic area. The audit tool was created using Google Forms as several of the younger group members were already familiar with it. It was also decided that the group would capture pictures of each observation location across 48 total stops (see Figure 2). Given the participants’ concerns over pollution, it was decided that air and water quality information would be captured simultaneously.

When considering how to implement the audit tool, the group took a practice walk a week before the planned walk and made route changes to improve data capture. At that point, it was decided that the larger group would be divided into two teams of seven people, covering 24 stops each. As there were 34 total questions/observations, it was decided that the survey should be split and collected by two team members to reduce the time each stop would take. Within the team, one team member would be assigned to survey A and another team member would be assigned to survey B. Survey A (https://forms.gle/ge8kPDRZBtQF65Jo6) contained eight questions for cleanliness/pollution and eight questions for traffic. Survey B (https://forms.gle/XRYHeEMTQVJFhBeT7) contained eight questions for neighborhood attractiveness/usefulness and ten questions for people/safety. Both surveys included a question about the stop number so that the data could be merged during analysis. To minimize the challenges of logging onto a survey, a QR code was created and printed out for each surveyor.

Team duties were pre-assigned. In addition to the surveyors, another individual would take photographs at each stop. A fourth individual would carry the map and direct the group to each spot as well as carry an air pollution sensor (continuously monitored during the walk using a Dylos Air Quality Monitors provided by the U.S. EPA). The remaining members would approach and ask residents if they were willing to provide a water sample.

### Phase 1: Early tool Implementation

While some group members had cell phones, most did not have unlimited data plans, so we provided them with 7-inch tablets connected to a Wi-Fi hotspot that we provided to use during the walk. The walk took place from 11 a.m. to 2 p.m. in mid-July. According to the historical weather data (Time and date, n.d.), the temperature on that day in Cincinnati was 82 degrees with 55% humidity (July 16, 2016).

Unfortunately, complete survey data was only collected at 32 of the planned 48 stops during the original walk due to a technology issue. To capture the missed observations, two surveyors collected data on the missing stops exactly one week later (July 23, 2016) during the same time frame while others analyzed water samples. According to the historical weather data, the temperature on that day in Cincinnati was 91 degrees with 59% humidity.

Water samples were collected at 18 stops during the walk. Group members tested the water for lead, pesticides, chlorine, nitrates, nitrites, copper, iron, bacteria, alkalinity, hardness, and pH using PurTest Home Drinking Water Analysis Test Kits provided by the U.S. EPA. Small (bacteria, mold, etc.) and large particles (pollen, etc.) were measured from the air quality monitors. Data were analyzed by time and the information was included in the final report/newsletter. No significant abnormalities were detected.

An undergraduate intern downloaded the resulting survey data, tabulated preliminary summaries, shared via paper, and discussed it with the larger group in Week 6. The group identified several concerns about their community, including broken or inadequate lighting, unkempt buildings, empty lots with a lot of trash, and graffiti on buildings. They were not overly concerned about traffic sound, lead levels in the water, or air particulate matter. The group also discussed ways to engage West End churches and other community members in proposed future clean-ups. The idea was to start a business that would provide stipends for participation in regular clean-ups. Supporting future assessments could be another role of stipend-paid community members. Finally, the group discussed the economics of a cleaner community and the benefits of getting the West End clean.

**Figure 3.**
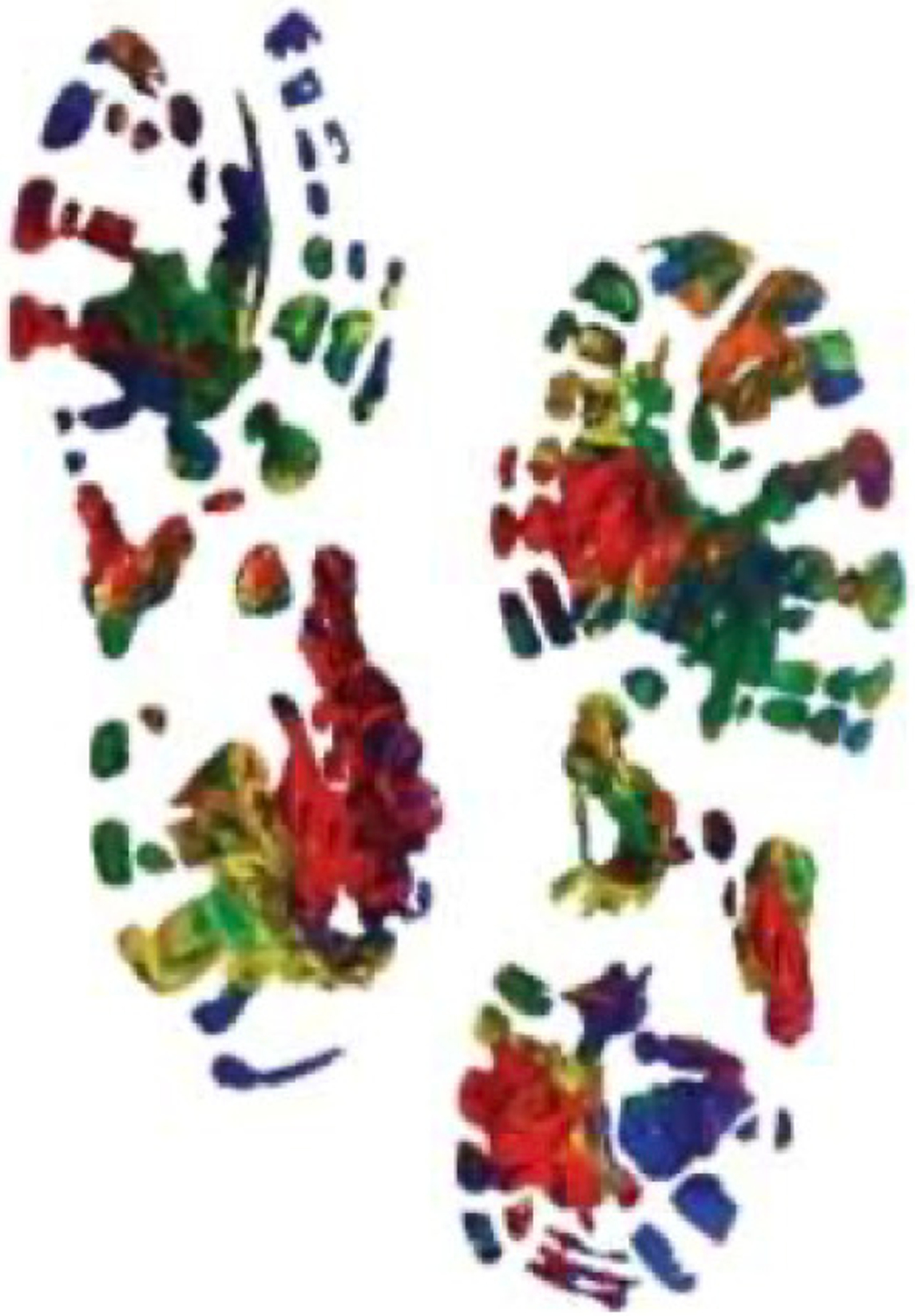
A Clean NeighborhoodIs saferElicits greater prideEncourages healthIncreases in beautyWelcomes economic growthIs our responsibility

A final report was created by the academic partner (MBK) and formatted as a newsletter to enable broad sharing with the community. Results were presented by select non-professional community scientists to the Community Council and via a set of posters (CM) at a neighborhood clean-up event one month after the program. Participants attended this event wearing t-shirts that were designed during the week 6 meeting (see Figure 3).

At the end of the week six meeting, participants were asked about their program experience. Participants indicated that they enjoyed learning about and sharing how the environment impacted health. They indicated that they had previously been largely unaware of its impact. Many, but not all, had enjoyed making observations and taking photos using the audit tool and felt more residents of their neighborhood would have benefitted from participation. The participants did express concern that most of their neighbors would not want to join a six-week program as they had. They wished the program could be modified so people could participate when it was more convenient for them.

### Phase 2: Tool Redevelopment

In response to our participants’ earlier feedback and agreeing that a six-week program may be prohibitive to the participation of many residents, in 2019 we considered how the audit tool could be optimized with the intent of making a tool that could be used independently by participants. The redesign was performed as part of the NIH R25 Science Education Program Award (SEPA) grant entitled “We Engage for Health (WE4H)” (https://weengage4health.life/). Briefly, by leveraging and growing the existing interdisciplinary community-academic partnership, the WE4H program’s goal is to improve the health literacy and health of individuals living in underserved communities. At that point, WE4H and community members had already co-developed an educational six-week program around community health (Health is Happen’ RAP) and planned to use community science projects to help the participants understand more about the environment they lived in. WE4H also had a training component, supporting summer interns in the work. One of the undergraduate summer interns selected (VH) had participated in the 2016 Phase 1 education program and audit development. As part of his summer project, he was tasked with helping modify the tool to enable broader participation.

There were several logistical changes made to the tool at that time. First, we moved away from using Google Forms to the more robust REDCap platform. REDCap was selected because we could not only capture survey answers but also simultaneously capture photos and geocoordinates to allow for easier geomapping and the creation of potential future Story Maps. In addition, we added consent language prior to the participant knowledge quiz. While our community science projects were not considered human subjects research by our IRB, we felt that including the consent statement was important because we had been working with our community members on the research process, highlighting participant consent as the key requirement of any human subject research.

To shorten the amount of time for the program, we developed educational text and included this text prior to each topic section rather than having in-person educational discussions. The purpose of this text was to increase participants’ knowledge about how the environment impacts health. Generally, three to five environmental health facts were included for each section.

The student intern was responsible for identifying these facts and modifying the related observation prompts included in the survey. As a result, topics were organized into five major themes: waste control, air pollution, outdoor spaces, housing, and traffic (see Table 3). Users would be prompted to make and record 38 observations in total across these topics. In addition to the environmental observations, we captured information on participants’ demographics (four questions) at the start of the survey. As participants were likely to have unlimited data phone plans, we designed the tool for personal mobile devices. Participants would also be encouraged to take photographs of their environments and respond to two questions. To ensure the privacy of non-participants, photos without faces of people were encouraged. Because we intended for the educational text to improve knowledge, we also included pre/post evaluation questions related to participant knowledge (10 questions each). Lastly, five questions designed to capture participant feedback and satisfaction would be given at two separate locations following observation recordings. The final complete audit survey included a total of 84 questions (see Table 3), and we expected our pilot participants to make and record their observations from at least two locations.

### Phase 2 Implementation

To evaluate the tool, we first invited past attendees from our We Engage 4 Health RAP Programs as well as the larger staff of our partnering organization. However, to maximize potential recruitment, we later expanded our recruitment efforts and invited individuals who had expressed interest in WE4H but had not yet completed a program. Invitations were sent by phone text, email, or via social media messaging within the networks of the community-academic partnership. Those willing to participate only had four days to make and record observations at two locations each. Given the time constraints of the student internship, we did not limit the use of the tool in the West End.

Twenty-six people made and recorded observations from at least one location using the audit tool. Most participants were female, black, and in the 18- to 34-year-old age group (see Table 4). While participants were asked to make observations at a minimum of two locations, 92% only made observations at one location. As only two participants made observations at two locations, we had only two post-tests and feedback forms completed. Most participants spent less than 10 minutes on the survey. Further, 77% of participants made observations on all five observational survey sections, and 15% of participants stopped after the first section. Only seven photos were provided by participants and most inappropriately included images of people. The 26 surveys which captured data were filled out in 18 different zip codes across the greater Cincinnati region.

With respect to the knowledge survey given before observations were made, the percent of correct results ranged from 23 to 85% across the questions (see Table 5). Questions most consistently answered correctly include the definition of research (Q3), the impact of overflowing garbage cans (Q4), and ease of breathing in hot weather (Q6). Because many of our participants were part of the larger WE4H, we expect that the concept of research and breathing on hot days may have been familiar as they had previously been discussed in our WE4H program. Notably, one of the questions with the lowest number of correct responses was the question concerning grassy areas and erosion/flooding (Q8). The quality and safety of housing questions scored similarly low (Q9; 23%). Thus, the pre-test scores suggest that there is room for improvement.

### Lessons Learned/Community Feedback

Once the walking audit was completed, the audit tool was presented at a community gathering of both academic and community partners and other miscellaneous stakeholders. Due to technical issues, the results were not available in time for the stakeholder meeting. At least five of the stakeholders present at the gathering participated/made observations using the tool. As most participants did not complete the survey at two locations, we did not capture feedback about the tool from the survey; all feedback was obtained at a community meeting where the intern shared his poster presentation on his effort at Seven Hills’ Community Center.

At this meeting, community stakeholders agreed that there is value in helping community members understand the impact of the environment on health, and an audit tool could have value in this effort. However, some of the pilot’s participants felt the audit tool was far too long, which is likely the reason most participants failed to repeat the assessment at a second location. Further, most participants present admitted that they had not read the educational text at the top of each section. Rather, they simply skipped it. The problem with skipping these sections is that the participants did not understand why they were making observations or how making observations benefited them personally. More engaging material was recommended when presenting the consent language, educational information, and instructions for the audit. One team member suggested that graphic-style short stories could be used to present the materials. Notably, graphic-style short stories co-designed with members of the West End community had been the foundation of the WE4H Health is Happen’ RAP program.

Another problem was the lack of clarity. Upon reviewing the questions, we found some discrepancies in interpretation. This suggests that despite engaging community members to help develop the survey (VH), additional validity and reproducibility studies were needed. As a group, we determined that including practice opportunities prior to initiating the actual assessment would be beneficial. The overall process was confusing for some, and a staff member of our partnering organization suggested that engaging videos would allow people to conveniently “train” prior to using the tool. Our community partners also identified a need for a larger context when using the audit tool. That is, the objective of the tool should be clearly stated. Engagement would be better if users were looking for data to answer a specific question or group of questions relevant to the community. They indicated that the use of interactive maps such as GIS or Story Maps would be beneficial to understanding the results. Finally, it was proposed that the tool be renamed to be more engaging to potential participants.

The student intern (VH) also presented a poster of his project at a research conference within our academic institution. There was an overall positive response to his work. The academics were especially impressed that the intern was from the community and had shared his results with his community first.

## Discussion

As people are often unaware that their local environments impact their health, we sought to provide community members with relevant and engaging education and opportunities to become non-professional community scientists within their own local communities. Working with a single predominately African American community, we partnered with community members who appreciated the need for greater environmental education. Over the course of several years, we generated two distinctly different community science audit tools, both of which engaged our community partners and served to increase participants’ and communities’ awareness of the environment around them and its role in their health. We garnered critical insights through community meetings; while neither of the tools were deemed optimal, there were important lessons learned from the process as well as from sharing the tool and data collected with community members. Our experiences helped strengthen our community-academic partnership and establish a route to create and tailor informal programming to meet local needs and engage people in community science with academic partners.

Looking back at our experiences, it is striking how different the two tools were even though both were focused on generating an audit tool to capture environmental factors in an urban environment. For the tool developed in Phase 1, community togetherness was an overarching goal. There was a sense of shared purpose, as the participants helped create the survey and understood its intent. Unfortunately, participants identified a barrier to greater participation, feeling that the program was too time intensive. Other community science projects also report time as a barrier (Roche et al., 2020). In the second phase of tool development, the ability to use the tool independently was an overarching goal. While that approach increased the ability for people to participate, the sense of purpose was lost on many. Indeed, a shared sense of purpose is required for a successful community science project (Coulson et al., 2021). In addition, the inclusion of a single community member in the initial development of the tool in Phase 2 was too limited, as we did not capture sufficient diversity of thought in the development phase. From these two projects, we have learned the importance of balance. Understanding both the limitations and strengths of each approach is essential to creating an optimal approach. When working with communities, multiple trials of tools or programs may be required to identify the best balance (D’Alonzo, 2010). Researchers must have tremendous patience and perseverance as there will likely be many “failures” before optimal programs can be designed (Westreicher et al., 2021). For example, very few participants completed the survey as intended when using our second tool. It was deemed too long, and some participants felt the questions were confusing. By typical research standards, this perturbation would be considered a failure. However, during the presentation of the tool to the community, critical insight on how this survey could be more engaging was provided. Moreover, the sharing of the environmental audit tool with the community provided increased awareness of the environment (for both phases) as well as reinforced the trust the community placed in the academic team. Thus, the role of the community-academic partnership was not simply to design the perfect tool or program on the first (or even second) try, but to create a process that contributes to ongoing trust—and authentically learning what works and what doesn’t.

One of our key takeaways was the importance of educating community partners. Community science has been recognized as a powerful tool to educate and inform the non-scientific community (de Sherbinin et al., 2021; Herodotou, 2018; Sprague et al., 2021). The challenge is finding the right approach. We learned that simply providing short, written passages about the topics of interest (as we did in the second phase) is often not enough, but relying solely on participatory approaches (as we did in the first phase) may be too time intensive for some. When working with community partners, it will be important to consider different learning styles and modes of engagement. Roche et al. (2020) concluded that considering the educational learning outcomes at the planning stage of the project is essential and that the use of co-creation approaches throughout the project can help to address issues of accessibility and inclusivity. The suggestion of co-creation approaches was brought up during the community meeting around the Phase 2 audit tool. Moving forward, we have leveraged co-creation and co-design activities to develop more engaging and relevant educational information.

We also found that it is important to have a clear shared purpose for any community-academic partnership projects (e.g., investment, motivation). The main goal of participating in community-based community science projects is to advance knowledge of societal relevance, raise public awareness, and promote problem-solving and actionable data (Kloetzer et al., 2021). When we switched to the independent focused audit tool in Phase 2, we found that many participants did not understand why the data captured by the audit tool was relevant to them. To ensure that the project goal is clear to participants, it will be essential to make it very easy for participants to understand the stated project goal. It’s essential to consider the optimal approach to present project goals; some participants may skip text, so an ideal approach may be to co-create the project goals statement and evaluate multiple presentation formats.

Beyond the project goals, sharing how the data could be relevant is essential. In our work, we captured information about the environment at multiple locations. By using geocoding, we can add richness and contextual relevance to the data captured while also minimizing participant burden (Thompson, 2016). Geographical Information Systems (GIS) has been shown to be highly useful by other environmental and health promotion programs (Barrie et al., 2019; Haklay & Francis, 2018; Jiao et al., 2015).

After obtaining feedback from our community partners, we have continued to refine this tool, building off what we learned from the previous phases. Moving onto the next phase of the tool, we are leveraging the lessons we have learned to date. Whether one has an established community partnership or is just starting out, we have found that it is critical to be mindful of the barriers to success (Gunnell et al., 2021; Hoover, 2016). At times in our audit tool development, we were beholden to deadlines which caused us to skip steps in the process (such as not pretesting in Phase 2, limiting collection days, and including more time for report/newsletter generation to permit community involvement in Phase 1). While no researcher, especially those embarking on a community-academic partnership, has a crystal ball, it is important to plan for contingencies and leverage the learning from other community researchers. Even for researchers who have a history in community-academic partnerships, starting new partnerships is always a challenge. Good communication is the hallmark of any community-academic partnership, but it is not sufficient (Agénor et al., 2018). The partnership will also require mutual trust and respect, both of which require time and patience to achieve (Fields et al., 2021; Mullins et al., 2020; Tisnado et al., 2010). One way to build and sustain trust is for the academic partner to *always* share information with the community. Researchers may be disinclined to share results from a project which did not turn out satisfactorily. However, it is important to share both the successes and the failures, as the community may have valuable insights into why something did not go as intended.

## Conclusions

Working through a community-academic partnership, we have raised awareness of the environment and its impact on health in an underserved community. By co-developing two distinct environmental audit tools, we have sought to educate community members about the local environment’s impact on their health. While the first two phases of the audit tool have not produced a useful “final” audit tool, the iterative process has provided key insights and emphasized the need for a balanced approach to co-development. Indeed, responsiveness to feedback and partnership with co-designers will ideally support the creation of more engaging programming, particularly in underserved communities with notable health disparities.

## Figures and Tables

**Table 1.** Phase 1 Audit Test Population Characteristics [N=14]

Characteristic	Grouping	Frequency
Age (years)	Under 18	37%
	18–35	21%
	36–55	21%
	56+	21%
Gender	Male	79%
	Female	21%
Ancestry	Black/African American	79%
	White/European	21%
Education	Less than high school diploma	42%
	High school diploma	14%
	2-year degree/some college	29%
	Bachelor’s degree or more	14%

**Table 2.** Environmental Exploration Program Structure: West End, Cincinnati 2016

Week	Phase	Meeting/Activity Mode
1	Education	Discussion: introductions, purpose of program; understanding how environment impacts health; impact of the environment on people and local economies
	Co-Design	Activity: Planning the remaining weeks.
2	Education	Discussion: air pollution, waste, water pollution, etc.; Flint, Michigan, and other environmental issues across the nation; using citizen science to inform understanding
	Co-design	Discussion: What information is needed to assess our neighborhood’s environmental health? Activity: Creating an audit tool
3	Co-design	Discussion: Audit team roles; learn how to use audit tool Activity: practice using audit tool at 2 locations, suggest changes, plan for neighborhood audit in week 4
4	Implementation	Activity: audit day - separate into 2 groups to capture date and collect samples
5	Implementation	Activity: test water samples, capture missing audit data
6	Implementation	Activity: review data, plan community report, plan and prepare for community sharing, create t-shirt logo/message, discuss lessons learned

**Table 3.** Phase 2 Walking Assessment Survey and Text Examples

Section	Survey Purpose	Category	# Questions (n=84)	Component Description or Example Educational Text
1	Consent to Participate	Being a Citizen Scientist	10	“Citizen science” is “scientific work undertaken by members of the general public, under the direction of professional scientists and scientific institutions”.
2	Evaluation	Demographics	4	Health risks and health-related behaviors differ by age, gender, race, and ethnicity.
3	Evaluation	Location Details	5	Cross streets, weather conditions
4	Evaluation	Pre-test	10	Knowledge of environmental health topics (given before the audit)
5	Walking Audit	Waste Control	9	Overflowing garbage cans are an ideal breeding ground for bacteria, insects, rats, and mice which cause illnesses.
6	Walking Audit	Air Pollution	6	Air pollutants can contain small and large particulate matter that can enter our lungs also make it harder for us to breath.
7	Walking Audit	Outdoor Spaces	13	People will use outdoor spaces more frequently and have greater pride in them when they are useful, appropriately lighted, clean, and maintained.
8	Walking Audit	Housing	5	Street address/neighborhood is a good predictor of heart disease; living in a disadvantaged neighborhood leads to a 50–80% increase in risk
9	Walking Audit	Traffic	5	City noise has been linked to impaired sleep and greater stress.
10	Evaluation	Photovoice-like	2	Photos can be used to help people describe their experiences, express your opinions, and share your voices (given after making observations at 2 locations).
11	Evaluation	Post-test	10	Knowledge of environmental health topics
12	Evaluation	Feedback	5	Understanding the value of the experience as citizen scientists (given after making observations at 2 locations)

**Table 4.** Phase 2 Audit Tool Test Population Characteristics [N=26]

Characteristic	Grouping	Frequency
Age(years)	Under 18	19%
	18–24	27%
	25–34	27%
	35–44	8%
	45–54	12%
	55–74	8%
Gender	Female	69%
	Male	31%
Ancestry	Black/African American	69%
	White/European	27%
	Other	8%
Ethnicity	Hispanic or Latino	4%

**Table 5.** Phase 2 Citizen Science Knowledge Survey

#	Questions (Correct Response)	Correct
1	Health is merely the absence of disease. (F)	58%
2	“Citizen science” is “scientific work undertaken by members of the general public under the direction of professional scientists. (T)	65%
3	Research is a way to make new observations, test new ideas and/or develop new tools. (T)	85%
4	Overflowing garbage cans are an ideal breeding ground for bacteria, insects, rats, and mice which cause illnesses. (T)	85%
5	Recycling is costly and has little value in disadvantaged neighborhoods. (F)	69%
6	It is easier to breath on sunny, high-temperature days when pollution from ozone is at its highest. (F)	81%
7	Trees, plants, and flowers help to remove pollutants from the air. (T)	69%
8	Unpaved grassy areas are at highest risk of erosion and flooding. (F)	23%
9	Improving the quality and safety of housing and outdoor areas in disadvantaged areas will do little to help residents take greater pride in their neighborhood and improve health. (F)	23%
10	City noise has been linked to impaired sleep and greater stress. (T)	65%

Response choices – true (T), false (F), I don’t know
